# Supplementary material for: Large electromagnetic field enhancement in plasmonic nanoellipse for tunable spaser based applications
Source: PLoS One. 2022 Mar 17;17(3):e0263630. doi: 10.1371/journal.pone.0263630 (PMC8929703; doi:10.1371/journal.pone.0263630)
Supplement: S1 Dataset — (PDF) [file pone.0263630.s001.pdf]

% Model: edfhy.mph

% Version: COMSOL 4.2.0.150 %

 - Volume Integration 2 (emw.Qrh/P0)

% freq emw.Qrh/P0 (m^2)

1.538E14 2.734204712561373E-16 1.5615217391304347E14 2.783929311467911E-16 1.5850434782608697E14 2.8340522148153297E-16 1.6085652173913044E14 2.8845824546451885E-16 1.632086956521739E14 2.935529220525863E-16 1.6556086956521738E14 2.9869018636126246E-16 1.6791304347826088E14 3.1053002978790275E-16 1.7026521739130434E14 3.2958852042286476E-16 1.726173913043478E14 3.5051257852391915E-16 1.749695652173913E14 3.73544246788023E-16 1.7732173913043478E14 3.98956931799361E-16 1.7967391304347825E14 4.270545127375866E-16 1.8202608695652175E14 4.581553293540818E-16 1.8437826086956522E14 4.927211465858607E-16 1.867304347826087E14 5.312454430138823E-16 1.890826086956522E14 5.742247028737649E-16 1.9143478260869566E14 6.22368850429655E-16 1.9378695652173912E14 6.764647163961606E-16 1.961391304347826E14 7.374679417678439E-16 1.984913043478261E14 8.065399743284172E-16 2.0084347826086956E14 8.849819890768102E-16 2.0319565217391303E14 9.744387306267064E-16 2.055478260869565E14 1.076841807067037E-15 2.079E14 1.1946617044724572E-15 2.1025217391304347E14 1.331047940764263E-15 2.1260434782608694E14 1.4897537576669278E-15 2.1495652173913044E14 1.6755652801411014E-15 2.173086956521739E14 1.8942426009420105E-15 2.1966086956521738E14 2.1532084642691717E-15 2.2201304347826088E14 2.4620375334028526E-15 2.2436521739130434E14 2.8336046260773285E-15 2.267173913043478E14 3.284714836343462E-15 2.290695652173913E14 3.8380799990507915E-15 2.3142173913043478E14 4.52496046173944E-15 2.3377391304347825E14 5.3876685254164836E-15 2.3612608695652175E14 6.477704493674705E-15 2.384782608695652E14 7.886179696351753E-15 2.408304347826087E14 9.736537328881547E-15 2.431826086956522E14 1.220866724697398E-14 2.4553478260869562E14 1.5518038587829065E-14 2.4788695652173912E14 2.0012134097948317E-14 2.502391304347826E14 2.6076303856195287E-14 2.5259130434782606E14 3.383641446994713E-14 2.5494347826086956E14 4.2518897563053126E-14 2.5729565217391303E14 4.9710990233005204E-14 2.596478260869565E14 5.1704966892286886E-14 2.62E14 4.732867525087632E-14 2.6435217391304347E14 3.926054825680164E-14 2.6670434782608694E14 3.098357816467487E-14 2.6905652173913044E14 2.4101064220985105E-14 2.714086956521739E14 1.879925281776871E-14 2.7376086956521738E14 1.4938756634590756E-14 2.7611304347826088E14 1.2040963742882738E-14 2.784652173913043E14 9.897632561118718E-15 2.808173913043478E14 8.26729987683621E-15 2.831695652173913E14 7.0102646642287705E-15 2.8552173913043475E14 6.033776241519437E-15 2.8787391304347825E14 5.2497922510862465E-15 2.9022608695652175E14 4.62697973651045E-15 2.925782608695652E14 4.110129643614849E-15 2.949304347826087E14 3.691152716327547E-15 2.972826086956521E14 3.3350391303924433E-15 2.996347826086956E14 3.0405234131105868E-15 3.019869565217391E14 2.7865065192028003E-15 3.043391304347826E14 2.572478704385779E-15 3.0669130434782606E14 2.386671265411333E-15 3.0904347826086956E14 2.2285939107007123E-15 3.11395652173913E14 2.092149317554379E-15 3.137478260869565E14 1.976887537723004E-15 3.161E14 1.88125132630247E-15 3.184521739130435E14 1.8025802240742206E-15 3.2080434782608694E14 1.744283476350063E-15 3.231565217391304E14 1.7022474998130114E-15 3.255086956521739E14 1.685005255258858E-15 3.278608695652174E14

1.692362590073976E-15 3.302130434782609E14 1.7325971473536798E-15 3.325652173913044E14
1.812311753504301E-15 3.349173913043478E14 1.929090806338175E-15 3.3726956521739125E14
2.065666931530405E-15 3.3962173913043475E14 2.186788919622802E-15 3.4197391304347825E14
2.2924585704909275E-15 3.4432608695652175E14 2.4297985010401352E-15 3.466782608695652E14
2.6264191899296722E-15 3.490304347826087E14 2.8306215613042122E-15 3.513826086956521E14
2.93930396724589E-15 3.537347826086956E14 2.9241069340686876E-15 3.560869565217391E14
2.864722940541199E-15 3.584391304347826E14 2.814398215854263E-15 3.6079130434782606E14
2.7673549415930563E-15 3.631434782608695E14 2.7400778178938117E-15 3.65495652173913E14
2.7876826701746592E-15 3.678478260869565E14 2.963492981566741E-15 3.702E14
3.2946169543779158E-15 3.725521739130435E14 3.823749387119725E-15 3.7490434782608694E14
4.588555479999505E-15 3.772565217391304E14 5.490189622803597E-15 3.796086956521739E14
6.259448797394151E-15 3.819608695652174E14 6.5446372720564676E-15 3.843130434782609E14
6.323962205227622E-15 3.866652173913043E14 6.0199253518337175E-15 3.890173913043478E14
5.934617480388726E-15 3.9136956521739125E14 6.232359213865283E-15 3.9372173913043475E14
6.9013892707823915E-15 3.9607391304347825E14 7.913822828791355E-15 3.9842608695652175E14
9.17254188296765E-15 4.007782608695652E14 1.0388123639581863E-14 4.031304347826086E14
1.1154635215677202E-14 4.054826086956521E14 1.1336433078904792E-14 4.078347826086956E14
1.100343002600785E-14 4.101869565217391E14 1.0535813456608598E-14 4.125391304347826E14
1.0188514319077616E-14 4.1489130434782606E14 9.906551548641635E-15 4.172434782608695E14
9.50495914380163E-15 4.19595652173913E14 8.911637404905461E-15 4.219478260869565E14
8.24807344172789E-15 4.243E14 7.677507261095702E-15 4.2665217391304344E14
7.287673499030405E-15 4.2900434782608694E14 7.039581017046666E-15 4.313565217391304E14
6.788318876380404E-15 4.337086956521739E14 6.400763272192094E-15 4.360608695652174E14
5.873277828599985E-15 4.384130434782608E14 5.327347341697632E-15 4.407652173913043E14
4.885998148239984E-15 4.431173913043478E14 4.5369057020931515E-15 4.4546956521739125E14
4.256966843409813E-15 4.4782173913043475E14 4.0249321981662795E-15 4.5017391304347825E14
3.851821645476037E-15 4.525260869565217E14 3.73885987697956E-15 4.548782608695652E14
3.69511849977577E-15 4.572304347826087E14 3.727100970576909E-15 4.595826086956521E14
3.8282433210125E-15 4.619347826086956E14 4.0085439581882046E-15 4.642869565217391E14
4.2661193588443276E-15 4.6663913043478256E14 4.616351616280328E-15 4.6899130434782606E14
5.059083997944674E-15 4.7134347826086956E14 5.5634015342851865E-15 4.73695652173913E14
6.0477996260837066E-15 4.760478260869565E14 6.433321633612109E-15 4.7839999999999994E14
6.6425069752579446E-15 4.8075217391304344E14 6.651372946941228E-15 4.8310434782608694E14
6.503319657764252E-15 4.854565217391304E14 6.284779985607743E-15 4.878086956521739E14
6.064648282310554E-15 4.901608695652174E14 5.8923380725990405E-15 4.925130434782608E14
5.790196005028915E-15 4.948652173913043E14 5.7561517732221055E-15 4.972173913043478E14
5.77236520083954E-15 4.9956956521739125E14 5.813480101492705E-15 5.0192173913043475E14
5.85960581972999E-15 5.0427391304347825E14 5.900800431924122E-15 5.066260869565217E14
5.935162247833843E-15 5.089782608695652E14 5.965851965748131E-15 5.113304347826087E14
5.9970591988308425E-15 5.136826086956521E14 6.031596735298263E-15 5.160347826086956E14
6.071093909685886E-15 5.183869565217391E14 6.114161134754353E-15 5.2073913043478256E14
6.157360331285404E-15 5.2309130434782606E14 6.196226733610493E-15 5.254434782608695E14
6.226018417743587E-15 5.27795652173913E14 6.2439944098125255E-15 5.301478260869565E14
6.2503896378545116E-15 5.3249999999999994E14 6.244475611672942E-15 5.3485217391304344E14

6.225829315570765E-15 5.3720434782608694E14 6.194133112534657E-15 5.395565217391304E14
6.149161242410425E-15 5.419086956521739E14 6.099315031575568E-15 5.442608695652174E14
6.039190767342184E-15 5.466130434782608E14 5.967296332512647E-15 5.489652173913043E14
5.8844960012622295E-15 5.513173913043478E14 5.79217382046712E-15 5.5366956521739125E14
5.692162440448081E-15 5.5602173913043475E14 5.586598106707247E-15 5.5837391304347825E14
5.479240143819506E-15 5.607260869565217E14 5.373294837603863E-15 5.630782608695652E14
5.270033698500798E-15 5.654304347826086E14 5.170271817045772E-15 5.677826086956521E14
5.074374908353927E-15 5.701347826086956E14 4.982320878305452E-15 5.72486956521739E14
4.893789687178872E-15 5.748391304347825E14 4.8116612762703575E-15 5.77191304347826E14
4.7350282506017804E-15 5.795434782608695E14 4.6614439606734036E-15 5.81895652173913E14
4.590466594244926E-15 5.842478260869565E14 4.521880459425874E-15 5.866E14
4.455628408899959E-15 5.889521739130435E14 4.391752251685366E-15 5.91304347826087E14
4.3303450948141175E-15 5.936565217391304E14 4.292356842362187E-15 5.960086956521739E14
4.259857937671284E-15 5.983608695652174E14 4.229433803238326E-15 6.007130434782608E14
4.201077978749178E-15 6.030652173913042E14 4.174768632209543E-15 6.054173913043478E14
4.150471479097373E-15 6.077695652173912E14 4.128142510732483E-15 6.101217391304348E14
4.107730416090636E-15 6.124739130434782E14 4.107077165638822E-15 6.148260869565218E14
4.120129147994384E-15 6.171782608695652E14 4.133579610670193E-15 6.195304347826086E14
4.147416440948375E-15 6.218826086956521E14 4.161628043409672E-15 6.242347826086956E14
4.176203312419869E-15 6.26586956521739E14 4.19113160568703E-15 6.289391304347825E14
4.206402718945655E-15 6.31291304347826E14 4.2220068617929656E-15 6.336434782608695E14
4.246002135400736E-15 6.35995652173913E14 4.2729473655450254E-15 6.383478260869565E14
4.299884276705133E-15 6.407E14 4.32681235948303E-15 6.430521739130435E14
4.353731055810904E-15 6.45404347826087E14 4.380639759087442E-15 6.477565217391304E14
4.407537814382177E-15 6.501086956521739E14 4.434424518715566E-15 6.524608695652172E14
4.461299121424358E-15 6.548130434782608E14 4.48695779645975E-15 6.571652173913042E14
4.5093070495022134E-15 6.595173913043478E14 4.531650278994932E-15 6.618695652173912E14
4.553986193133074E-15 6.642217391304348E14 4.5763134789864255E-15 6.665739130434782E14
4.5986308036601215E-15 6.689260869565218E14 4.620936815611026E-15 6.712782608695652E14
4.643230146130127E-15 6.736304347826086E14 4.665509410998112E-15 6.759826086956521E14
4.687773212329121E-15 6.783347826086956E14 4.7094785582568284E-15 6.80686956521739E14
4.726653102574966E-15 6.830391304347825E14 4.743802052421844E-15 6.85391304347826E14
4.760924222453709E-15 6.877434782608695E14 4.778018439385098E-15 6.90095652173913E14
4.795083544952383E-15 6.924478260869565E14 4.812118399080534E-15 6.948E14
4.829121883257321E-15 6.971521739130435E14 4.846092904115239E-15 6.995043478260869E14
4.863030397227125E-15 7.018565217391304E14 4.879933331110959E-15 7.042086956521739E14
4.89648082158175E-15 7.065608695652172E14 4.911496159854625E-15 7.089130434782608E14
4.926449085367016E-15 7.112652173913042E14 4.9413388807132574E-15 7.136173913043478E14
4.956164900390607E-15 7.159695652173912E14 4.9709265759540384E-15 7.183217391304348E14
4.9856234212830745E-15 7.206739130434782E14 5.000255037943689E-15 7.230260869565216E14
5.014821120623914E-15 7.253782608695651E14 5.0293214626222154E-15 7.277304347826086E14
5.0437559613629636E-15 7.300826086956521E14 5.058124623913073E-15 7.324347826086956E14
5.071292332048585E-15 7.347869565217391E14 5.082580941200683E-15 7.371391304347825E14
5.0937946014211844E-15 7.39491304347826E14 5.1049340613552236E-15 7.418434782608695E14

5.116000201045861E-15 7.44195652173913E14 5.126994035918844E-15 7.465478260869565E14
5.1379167204920814E-15 7.489E14 5.148769551772667E-15 7.512521739130434E14
5.159553972299503E-15 7.536043478260869E14 5.170271572788881E-15 7.559565217391304E14
5.180924094344291E-15 7.583086956521739E14 5.191513430185868E-15 7.606608695652174E14
5.202041626861926E-15 7.630130434782608E14 5.210728051556604E-15 7.653652173913042E14
5.218280228684889E-15 7.677173913043478E14 5.225759844555996E-15 7.700695652173912E14
5.233169710033187E-15 7.724217391304348E14 5.240512775407693E-15 7.747739130434782E14
5.247792126567978E-15 7.771260869565216E14 5.255010980424907E-15 7.794782608695651E14
5.262172679581619E-15 7.818304347826086E14 5.2692806862415095E-15 7.841826086956521E14
5.27633857535056E-15 7.865347826086956E14 5.283350026981618E-15 7.888869565217391E14
5.290318817969317E-15 7.912391304347825E14 5.297248812811652E-15 7.93591304347826E14
5.304143953863228E-15 7.959434782608695E14 5.308245377656538E-15 7.98295652173913E14
5.311534736127588E-15 8.006478260869565E14 5.314812927386638E-15 8.029999999999999E14
5.318084010643556E-15 8.053521739130434E14 5.321352050748015E-15 8.077043478260869E14
5.3246211067905974E-15 8.100565217391304E14 5.3278952204442515E-15 8.124086956521739E14
5.331178404108392E-15 8.147608695652174E14 5.3344746289214694E-15 8.171130434782608E14
5.337787812707736E-15 8.194652173913042E14 5.341121807931941E-15 8.218173913043478E14
5.3444803897298155E-15 8.241695652173912E14 5.347867244089839E-15 8.265217391304348E14
5.3512859562569135E-15 8.288739130434782E14 5.3547399994309425E-15 8.312260869565216E14
5.362954181747569E-15 8.335782608695651E14 5.373361820733389E-15 8.359304347826086E14
5.3838195041903906E-15 8.382826086956521E14 5.3943305411944626E-15 8.406347826086956E14
5.404898079589534E-15 8.42986956521739E14 5.4155250942786135E-15 8.453391304347825E14
5.426214376219051E-15 8.47691304347826E14 5.436968522181404E-15 8.500434782608695E14
5.447789925332297E-15 8.52395652173913E14 5.458680766689754E-15 8.547478260869565E14
5.469643007495356E-15 8.570999999999999E14 5.48067838253719E-15

% Model: edfhx.mph

% Version: COMSOL 4.2.0.150 %

Surface Integration

(emw.normEfar^2/E0^2)

% freq emw.normEfar^2/E0^2 (m^2)

1.538E14 6.334710167409506E-30 1.5615217391304347E14 6.752301511373678E-30
1.5850434782608697E14 7.191261870009552E-30 1.6085652173913044E14 7.65240877260748E-30
1.632086956521739E14 8.136583098611889E-30 1.6556086956521738E14 8.644649638140842E-30
1.6791304347826088E14 9.432086875694065E-30 1.7026521739130434E14 1.0533922810946938E-29
1.726173913043478E14 1.1768253850546536E-29 1.749695652173913E14 1.3207052402245022E-29
1.7732173913043478E14 1.493727742715192E-29 1.7967391304347825E14 1.7026161954808601E-29
1.8202608695652175E14 1.9531762518618022E-29 1.8437826086956522E14 2.2554988571543709E-29 1.867304347826087E14 2.622863330913669E-29 1.890826086956522E14 3.0718872415127947E-29

1.9143478260869566E14 3.6286804462325574E-29 1.9378695652173912E14 4.328647843219447E-29
1.961391304347826E14 5.22192308770148E-29 1.984913043478261E14 6.380400137663435E-29
2.0084347826086956E14 7.906274068677509E-29 2.0319565217391303E14 9.944770178568663E-29
2.055478260869565E14 1.2691883790771174E-28 2.079E14 1.638207129474048E-28
2.1025217391304347E14 2.117594386513135E-28 2.1260434782608694E14 2.679775761595228E-28
2.1495652173913044E14 3.1898526260121274E-28 2.173086956521739E14 3.4003785749095455E-28
2.1966086956521738E14 3.1622435590320407E-28 2.2201304347826088E14 2.6174442801383477E-28 2.2436521739130434E14 2.0208660671838174E-28 2.267173913043478E14 1.5171706270037068E-28 2.290695652173913E14 1.1349798000989746E-28 2.3142173913043478E14 8.55400664857744E-29
2.3377391304347825E14 6.52062922716698E-29 2.3612608695652175E14 5.0380916043649123E-29
2.384782608695652E14 3.9290454815684656E-29 2.408304347826087E14 3.0859280208947997E-29
2.431826086956522E14 2.4348542517518223E-29 2.4553478260869562E14 1.9308670357675944E-29
2.4788695652173912E14 1.5315376246741622E-29 2.502391304347826E14 1.2113023262128729E-29
2.5259130434782606E14 9.549168416903318E-30 2.5494347826086956E14 7.506076202142184E-30
2.5729565217391303E14 5.8656973921804926E-30 2.596478260869565E14 4.5833582158046404E-30
2.62E14 3.625656332850908E-30 2.6435217391304347E14 2.9381918888711704E-30
2.6670434782608694E14 2.4808865678027354E-30 2.6905652173913044E14 2.1653364188050874E-30 2.714086956521739E14 1.9227512352543084E-30 2.7376086956521738E14 1.7795274518692832E-30 2.7611304347826088E14 1.795696554006094E-30 2.784652173913043E14 2.0432813891716224E-30 2.808173913043478E14 2.572209128707223E-30 2.831695652173913E14 3.4316134872869954E-30
2.8552173913043475E14 4.665027946344883E-30 2.8787391304347825E14 6.355203143234696E-30
2.9022608695652175E14 8.513470467645271E-30 2.925782608695652E14 1.12280748919038E-29
2.949304347826087E14 1.4445309441704566E-29 2.972826086956521E14 1.8473636352132057E-29
2.996347826086956E14 2.3454233329764087E-29 3.019869565217391E14 2.9562975282992635E-29
3.043391304347826E14 3.5718893346103716E-29 3.0669130434782606E14 3.9869041042051675E-29
3.0904347826086956E14 3.9396992849013876E-29 3.11395652173913E14 3.4195349062888546E-29
3.137478260869565E14 2.7309792271293764E-29 3.161E14 2.1883086992450195E-29
3.184521739130435E14 1.9128679925896024E-29 3.2080434782608694E14 1.926004267037372E-29
3.231565217391304E14 2.2085754828749688E-29 3.255086956521739E14 2.7591366708108693E-29
3.278608695652174E14 3.620140107932983E-29 3.302130434782609E14 4.86886566088109E-29
3.325652173913044E14 6.618040729414263E-29 3.349173913043478E14 9.107764868892948E-29
3.3726956521739125E14 1.2514671290135442E-28 3.3962173913043475E14 1.7218540895547483E-28 3.4197391304347825E14 2.342050155517342E-28 3.4432608695652175E14 3.0571151153166834E-28 3.466782608695652E14 3.723373724329093E-28 3.490304347826087E14 4.067331029047636E-28
3.513826086956521E14 3.9971107200843437E-28 3.537347826086956E14 3.673249866697371E-28
3.560869565217391E14 3.2626421073076244E-28 3.584391304347826E14 2.803801065028436E-28
3.6079130434782606E14 2.3228655936915456E-28 3.631434782608695E14 1.888467331268208E-28
3.6549565217391300E14 1.5224568458102018E-28 3.678478260869565E14 1.2261290795849053E-28
3.702E14 9.968796996177245E-29 3.725521739130435E14 8.137451316133865E-29
3.7490434782608694E14 6.648293880912903E-29 3.772565217391304E14 5.479897743566653E-29
3.796086956521739E14 4.543715654441689E-29 3.819608695652174E14 3.757652979864918E-29
3.843130434782609E14 3.1079227276875885E-29 3.866652173913043E14 2.591944297554701E-29
3.890173913043478E14 2.1428361996355276E-29 3.9136956521739125E14 1.7537268267248665E-29
3.9372173913043475E14 1.4546311558263561E-29 3.9607391304347825E14 1.2094714191211767E-

29 3.9842608695652175E14 1.0124134018150177E-29 4.007782608695652E14 8.72253100407987E-30
4.031304347826086E14 7.970218181345458E-30 4.054826086956521E14 7.731318865462214E-30
4.078347826086956E14 8.01903461486166E-30 4.101869565217391E14 8.8155658542606E-30
4.125391304347826E14 1.0138680464878692E-29 4.1489130434782606E14 1.2152959309510368E-29
4.172434782608695E14 1.5100990073554268E-29 4.19595652173913E14 1.9359727087359408E-29
4.219478260869565E14 2.557873497538787E-29 4.243E14 3.4779080471796205E-29
4.2665217391304344E14 4.8335043043291296E-29 4.2900434782608694E14 6.740116586099846E-29
4.313565217391304E14 9.101735780243321E-29 4.337086956521739E14 1.141425568230854E-28
4.360608695652174E14 1.2847683619993505E-28 4.384130434782608E14 1.2915437515274617E-28
4.407652173913043E14 1.185963419292766E-28 4.431173913043478E14 1.0357778615883898E-28
4.4546956521739125E14 8.785011844905383E-29 4.4782173913043475E14 7.337464162945248E-29
4.5017391304347825E14 6.120492716926888E-29 4.525260869565217E14 5.128093085697114E-29
4.548782608695652E14 4.3141295666192915E-29 4.572304347826087E14 3.648942068862059E-29
4.595826086956521E14 3.110440027659804E-29 4.619347826086956E14 2.716792773541792E-29
4.642869565217391E14 2.4233806990824547E-29 4.6663913043478256E14 2.225278755616305E-29
4.6899130434782606E14 2.126943850072346E-29 4.7134347826086956E14 2.13625515819133E-29
4.73695652173913E14 2.2542191525988514E-29 4.760478260869565E14 2.4628781122457904E-29
4.7839999999999994E14 2.723176821473036E-29 4.8075217391304344E14 2.9751507804188985E-29
4.8310434782608694E14 3.1614809974376846E-29 4.854565217391304E14 3.2591222197876707E-29
4.878086956521739E14 3.277577760088801E-29 4.901608695652174E14 3.24619867361964E-29
4.925130434782608E14 3.196539002808496E-29 4.948652173913043E14 3.1475231180653505E-29
4.972173913043478E14 3.1044753373390077E-29 4.9956956521739125E14 3.0657954837389536E-29
5.0192173913043475E14 3.0246972466656566E-29 5.0427391304347825E14 2.979861965131479E-29
5.066260869565217E14 2.9309708910215364E-29 5.089782608695652E14 2.8771087766355E-29
5.113304347826087E14 2.819869064499791E-29 5.136826086956521E14 2.760690813829662E-29
5.160347826086956E14 2.697056074345817E-29 5.183869565217391E14 2.6300652670937576E-29
5.2073913043478256E14 2.560303040967107E-29 5.2309130434782606E14 2.487655734951312E-29
5.254434782608695E14 2.4143267253923184E-29 5.27795652173913E14 2.3438585326230906E-29
5.301478260869565E14 2.2702971231476823E-29 5.3249999999999994E14 2.1941071473892978E-29
5.3485217391304344E14 2.1160294237918724E-29 5.3720434782608694E14 2.0369629034034016E-
29 5.395565217391304E14 1.9578627982494227E-29 5.419086956521739E14 1.8843975311496117E-
29 5.442608695652174E14 1.812907444809821E-29 5.466130434782608E14 1.742925791324021E-29
5.489652173913043E14 1.6751084628962507E-29 5.513173913043478E14 1.6100282580852397E-29
5.5366956521739125E14 1.5481553539341268E-29 5.5602173913043475E14 1.489838079533568E-29
5.5837391304347825E14 1.4404850907530274E-29 5.607260869565217E14 1.395312303193437E-29
5.630782608695652E14 1.3536268379499758E-29 5.654304347826086E14 1.3149981294596594E-29
5.677826086956521E14 1.2789211622096332E-29 5.701347826086956E14 1.2448560475931531E-29
5.72486956521739E14 1.2122700469860706E-29 5.748391304347825E14 1.185106876486783E-29
5.77191304347826E14 1.1609622895168409E-29 5.795434782608695E14 1.137121701069695E-29
5.81895652173913E14 1.1136780273894875E-29 5.842478260869565E14 1.0908036895947717E-29
5.866E14 1.0687131380229351E-29 5.889521739130435E14 1.0476348750789213E-29
5.91304347826087E14 1.0277923124262189E-29 5.936565217391304E14 1.0204930652558043E-29
5.960086956521739E14 1.0162890790040474E-29 5.983608695652174E14 1.0134433896240554E-29
6.007130434782608E14 1.0120300454770571E-29 6.030652173913042E14 1.0121110129963971E-29

6.054173913043478E14 1.013738213206613E-29 6.077695652173912E14 1.0169553192545279E-29
6.101217391304348E14 1.0217993090729804E-29 6.124739130434782E14 1.0362588534072439E-29
6.148260869565218E14 1.0574736116840307E-29 6.171782608695652E14 1.0794899481697935E-29
6.195304347826086E14 1.1023187438122658E-29 6.218826086956521E14 1.1259709951689814E-29
6.242347826086956E14 1.1504578105675901E-29 6.26586956521739E14 1.1757904057748618E-29
6.289391304347825E14 1.2019800992264055E-29 6.31291304347826E14 1.2290383068544928E-29
6.336434782608695E14 1.2580841058125687E-29 6.35995652173913E14 1.288126691357324E-29
6.383478260869565E14 1.318727155114471E-29 6.407E14 1.3498912647028237E-29
6.430521739130435E14 1.381624740561299E-29 6.45404347826087E14 1.4139332523087306E-29
6.477565217391304E14 1.4468224150433346E-29 6.501086956521739E14 1.4802977855883288E-29
6.524608695652172E14 1.5143648586903543E-29 6.548130434782608E14 1.5475606082695902E-29
6.571652173913042E14 1.5772179643920922E-29 6.595173913043478E14 1.6073058911902003E-29
6.618695652173912E14 1.6378273773610258E-29 6.642217391304348E14 1.6687853552635544E-29
6.665739130434782E14 1.7001826989921346E-29 6.689260869565218E14 1.7320222225089352E-29
6.712782608695652E14 1.7643066778446424E-29 6.736304347826086E14 1.7970387533813358E-29
6.759826086956521E14 1.8302210722289822E-29 6.783347826086956E14 1.8631723478641392E-29
6.80686956521739E14 1.890793402208722E-29 6.830391304347825E14 1.9187050669425035E-29
6.85391304347826E14 1.9469081440290672E-29 6.877434782608695E14 1.9754033961731047E-29
6.90095652173913E14 2.0041915471731565E-29 6.924478260869565E14 2.033273282420087E-29
6.948E14 2.0626492495508296E-29 6.971521739130435E14 2.0923200592670273E-29
6.995043478260869E14 2.1222862863265005E-29 7.018565217391304E14 2.1525484707183184E-29
7.042086956521739E14 2.1821864171216024E-29 7.065608695652172E14 2.207779370993837E-29
7.089130434782608E14 2.233533409762124E-29 7.112652173913042E14 2.2594476954911413E-29
7.136173913043478E14 2.2855213928722418E-29 7.159695652173912E14 2.3117536722385145E-29
7.183217391304348E14 2.3381437127117837E-29 7.206739130434782E14 2.3646907054764322E-29
7.230260869565216E14 2.3913938571726158E-29 7.253782608695651E14 2.418252393400744E-29
7.277304347826086E14 2.4452655623292663E-29 7.300826086956521E14 2.4724326383920294E-29
7.324347826086956E14 2.4983217049712108E-29 7.347869565217391E14 2.5220387086728665E-29
7.371391304347825E14 2.545839772139459E-29 7.39491304347826E14 2.569724106496775E-29
7.418434782608695E14 2.593690971204361E-29 7.44195652173913E14 2.617739676993549E-29
7.465478260869565E14 2.6418695887302956E-29 7.489E14 2.666080128183116E-29
7.512521739130434E14 2.690370776677262E-29 7.536043478260869E14 2.714741077612397E-29
7.559565217391304E14 2.739190638823936E-29 7.583086956521739E14 2.763719134764459E-29
7.606608695652174E14 2.7883263084856E-29 7.630130434782608E14 2.8099056793315016E-29
7.653652173913042E14 2.829644178802828E-29 7.677173913043478E14 2.849377224094929E-29
7.700695652173912E14 2.8691046223573785E-29 7.724217391304348E14 2.888826248019809E-29
7.747739130434782E14 2.908542041564723E-29 7.771260869565216E14 2.9282520079572506E-29
7.794782608695651E14 2.9479562147258277E-29 7.818304347826086E14 2.967654789688769E-29
7.841826086956521E14 2.987347918325253E-29 7.865347826086956E14 3.0070358407919097E-29
7.888869565217391E14 3.0267188485870403E-29 7.912391304347825E14 3.04639728086846E-29
7.93591304347826E14 3.066071520434627E-29 7.959434782608695E14 3.084529755750275E-29
7.98295652173913E14 3.102653493077622E-29 8.006478260869565E14 3.1207950391253657E-29
8.029999999999999E14 3.1389554600625525E-29 8.053521739130434E14 3.1571358442235513E-29
8.077043478260869E14 3.1753372984763035E-29 8.100565217391304E14 3.193560944476272E-29

8.124086956521739E14 3.2118079148231336E-29 8.147608695652174E14 3.2300793491406037E-29
8.171130434782608E14 3.2483763900988223E-29 8.194652173913042E14 3.2667001793999675E-29
8.218173913043478E14 3.285051853748864E-29 8.241695652173912E14 3.303432540829441E-29
8.265217391304348E14 3.3218433553101743E-29 8.288739130434782E14 3.3402853948979147E-29
8.312260869565216E14 3.363122516553922E-29 8.335782608695651E14 3.388013591690138E-29
8.359304347826086E14 3.412981729606037E-29 8.382826086956521E14 3.4380276915347153E-29
8.406347826086956E14 3.4631521840543713E-29 8.42986956521739E14 3.488355852266735E-29
8.453391304347825E14 3.5136392731407563E-29 8.47691304347826E14 3.539002949057611E-29
8.500434782608695E14 3.564447301589004E-29 8.52395652173913E14 3.589972665541986E-29
8.547478260869565E14 3.6155792832964495E-29 8.570999999999999E14 3.6412672994662514E-29
